# The Foreign Journals: Medical Statistics

**Published:** 1839-10

**Authors:** 


### MEDICAL STATISTICS.

### *On Longevity and Mortality in Prussia.* By Dr. HOFFMAN.

DURING the eighteen years, commencing 1st January, 1820, and ending 31st December, 1837, the mortality in 12,626,379, being the average population of Prussia during that period, amounted to 6,653,167. Of this number, 149,058 attained the age of from eighty to eighty-five years; 67,754 that of from eighty-five to ninety years; and 31,516 attained an age beyond ninety years. Thus, 248,328 individuals, or 3·732 per cent. of the population attained an age beyond eighty.

During the same period, the births amounted to 9,236,107. Of this number, 319,243 were stillborn; 1,577,018 more died before the completion of the first year; 751,737 died during the second and third years; 305,237, during the fourth and fifth years; 171,808, during the sixth and seventh; 154,124, during the eighth, ninth, and tenth; and 123,693, during the eleventh, twelfth, thirteenth, and fourteenth years. *Medicinische Zeitung.* No. iv. 1839.

